# Functional Analysis of Wheat NAC Transcription Factor, *TaNAC069*, in Regulating Resistance of Wheat to Leaf Rust Fungus

**DOI:** 10.3389/fpls.2021.604797

**Published:** 2021-03-15

**Authors:** Yanjun Zhang, Huaimin Geng, Zhongchi Cui, Haiyan Wang, Daqun Liu

**Affiliations:** ^1^College of Plant Protection, Hebei Agricultural University/Technological Innovation Center for Biological Control of Crop Diseases and Insect Pests of Hebei Province, Baoding, China; ^2^Graduate School of Chinese Academy of Agricultural Sciences, Beijing, China

**Keywords:** RNA-seq, *Puccinia triticina*, NAC transcription factor, clone, VIGS

## Abstract

NAC transcription factors are one of the largest transcription factor families having functions in a variety of stress responses. Few NACs have been reported for interactions between wheat and the wheat rust fungus *Puccinia triticina* (*Pt*). In this study, based on analysis of RNA-seq data from wheat line TcLr19 inoculated by *Pt*, the NAC transcription factor *TaNAC069* was cloned from wheat, and its transcriptional activity and homologous dimer formation were verified. Quantitative real-time PCR analysis showed that the expression of *TaNAC069* was induced by *Pt* and associated signaling molecules. To further characterize the function of the *TaNAC069* gene in wheat resistance to *Pt*, virus-induced gene silencing (VIGS) was utilized, and it revealed that *Pt* resistance in *TaNAC069*-silenced plants was significantly reduced. Potential interaction targets of TaNAC069 from wheat and *Pt* were screened and identified by yeast two-hybrid technology. Eukaryotic elongation factor *eEF1A*, CBSX3 protein, and cold acclimation protein WCOR410c were screened by yeast one-hybrid technology. The results indicate that the *TaNAC069* gene plays a positive regulatory role in wheat resistance to *Pt*, laying a good foundation to analyze the molecular mechanisms of *TaNAC069* and its functional role in wheat resistance to *Pt*.

## Introduction

At the transcriptional level, controlling gene expression is key to regulate many plant cell responses, including normal signal transduction and cell morphological changes in response to stress (Hu et al., 2010). Many studies have shown that transcription factors regulate the expression of stress-related genes to activate or repress the expression of target genes involved in stress responses (Erpen et al., 2018). NAC transcription factors are one of the largest families of plant-specific transcription factors that respond to biotic and abiotic stresses by participating in plant stress signal transduction pathways and regulating the expression of downstream target genes (Zhang et al., 2018). A common feature of NAC transcription factor family members is that they contain a conserved NAC domain in the N-terminus, which is usually divided into five subdomains—A, B, C, D, and E—as well as a highly variable regulatory domain in the C-terminus (Shao et al., 2015). The C-terminus of NAC is rich in serine, threonine, proline, and some acidic amino acids and can activate or repress the expression of downstream target genes (Olsen et al., 2005). Additionally, the C-terminus may have transcriptional activity, containing different motifs having specific functions.

NAC transcription factors generally function by forming homodimers or heterodimers of NAC proteins. Different NAC proteins have different biological functions (Ooka et al., 2003). Many reports have demonstrated that NAC transcription factors play important roles in enhancing drought and salt resistance (Nguyen et al., 2019), improving tolerance to dehydration and high salt stress (Nakashima et al., 2007), enhancing tolerance to *Erysiphe cichoracearum* and *Phytophthora parasitica* var. *nicotianae* (Zhu et al., 2012), and regulating plant defense responses against fungal and bacterial pathogens (Wang et al., 2007).

Many studies have shown that NAC proteins play significant roles in different signaling pathways. NAC proteins respond to biotic and abiotic stresses through regulation of downstream gene expression or through interaction with other proteins (Wu et al., 2018). *GhirNAC2* plays a positive role in improving drought tolerance in cotton by regulating abscisic acid (ABA) biosynthesis (Shang et al., 2020). The NAC transcription factor CrNAC036 cooperates with CrMYB68 transcription factors to repress ABA biosynthesis by negatively regulating the expression of the *CrNCED5* (9-*cis*-epoxycarotenoid dioxygenase 5) gene in citrus fruit (Zhu et al., 2020). TaNACL-D1 (*Triticum aestivum* NAC-like transcription factor) can interact with *TaFROG* (*T. aestivum* fusarium resistance orphan gene) and positively enhances wheat resistance to Fusarium head blight disease (Perochon et al., 2019). The expression levels of the *TaNAC4* and *TaNAC8* genes were significantly up-regulated during incompatible interactions between wheat and *Pst* (*Puccinia striiformis* f. sp. *tritici*) (Xia et al., 2010).

Wheat leaf rust is a fungal disease caused by the obligate, biotrophic plant pathogen *Puccinia triticina* (*Pt*), which cannot grow without living hosts. It is one of the most widespread and destructive diseases of wheat worldwide, causing significant yield losses in major wheat-growing regions (Kolmer, 1996). Frequent variations in virulence genes of *Pt* yield new virulent species that can evade detection by wheat resistance genes, leading to the loss of rust-resistant wheat cultivars (Kolmer et al., 2020), which is likely to result in the outbreak of leaf rust. Thus, the excavation, screening, and identification of wheat resistance genes is crucial to understanding the molecular mechanisms underlying resistance against the wheat leaf rust pathogen. Wheat leaf rust resistance gene, *Lr19*, originally identified in *Agropyron elongatum*, on the 7DL chromosome, provides effective resistance to most pathotypes of *Pt* throughout the growth period of wheat (Sarma and Knott, 1966; Ordoñez and Kolmer, 2008). Recently, 453 NAC proteins in polyploid wheat were identified (Borrill et al., 2017). However, the specific functions and characteristics of NAC transcription factors are unknown, especially those involved in wheat resistance against *Pt.* In our previous study, based on the analysis of RNA-seq data of wheat, TcLr19, a NAC transcription factor induced by *Pt* (Traes_5DL_D1D0CA79E), was screened by combining differential gene expression and functional annotation (Zhang et al., 2020). In this study, we cloned the NAC transcription factor, aimed to identify its function in wheat resistance to *Pt* using barley stripe mosaic virus (BSMV)-induced gene silencing, and screened for interacting protein targets by yeast two-hybrid and yeast one-hybrid technologies, laying a good foundation to analyze the molecular mechanism of this NAC transcription factor underlying wheat resistance to *Pt*.

## Materials and Methods

### Plant Materials and Inoculation System

The near-isogenic lines TcLr19, Thatcher, Jinhe, and *Pt* race 07-10-426-1 (PHNT) were used for gene expression and functional analyses. TcLr19, which expresses a typical hypersensitive response (HR) (scale 0), and Thatcher, which is susceptible to PHNT (scale 4), were inoculated with *Pt* race PHNT according to Roelfs’ standard (Roelfs et al., 1984). Leaf, stem, and root samples were taken from PHNT and mock inoculated (with sterile distilled water) wheat plants at 0, 6, 12, 24, 48, 72, 96, 144, and 168 h post-inoculation (hpi) for total RNA extraction. Then, 0.1 mmol/L ABA, salicylic acid (SA), ethephon (ETH), and methyl jasmonate (MeJA) were sprayed on the surface of leaves of 7-day-old wheat seedlings at the primary leaf stage, respectively. Wheat leaf samples were harvested at 0, 24, 48, 72, 96, and 120 h post-treatment (hpt) for total RNA extraction.

### Cloning of the *TaNAC069* Gene and *TaNAC069* Promoter and Bioinformatics Analysis

A pair of gene-specific primers, *TaNAC069*-F and *TaNAC069*-R (Supplementary Table 1), was designed based on conserved regions of the NAC sequence mapping in the RNA-seq data. A mixture of RNA isolated at different time points from wheat leaves infected by *Pt* was used as the template in reverse transcription polymerase chain reaction (RT-PCR) to obtain fragments of *TaNAC069*. The conserved domains of *TaNAC069* gene were deduced using NCBI (http://www.ncbi.nlm.nih.gov/Structure/cdd/wrpsb.cgi). CLUSTALW was used to create multiple alignments. MEGA5 (http://www.megasoftware.net) was used to generate phylogenetic trees.

Searching sequences upstream of *TaNAC069* genes in the wheat database (http://plants.ensembl.org/Taestivum/Info/ Index), an approximately 2.0-kb sequence of the homologous sequence of *TaNAC069* on 5D was intercepted from each gene and used to design primers pTaNAC069-F and pTaNAC069-R (Supplementary Table 1). Genomic DNA of TcLr19 was used as template, and RT-PCR was performed to generate the promoter of the *TaNAC069* gene. The *cis*-acting elements of the promoter were analyzed using the PlantCARE (http://bioinformatics.psb.ugent.be/ webtools/plantcare/html/) and PLACE (http://www. dna.affrc.go.jp/PLACE/index.html) databases (Sun et al., 2015).

### Expression Analysis of *TaNAC069* by qPCR

The specific primers qTaNAC069-F and qTaNAC069-R were used to investigate the expression level of *TaNAC069*. Wheat glyceraldehyde-3-phosphate dehydrogenase (GAPDH, GenBank: AF251217) was used as an endogenous control as previously described by Gao et al. (2015). Quantitative real-time PCR (qPCR) was performed using TransStart^®^ Top Green qPCR SuperMix (TransGen Biotech, China) according to the manufacturer’s instructions. All qPCR experiments were performed on a LightCycler^®^ 96 Real-Time PCR System (Roche, Switzerland), and relative gene expression was calculated using the 2^–ΔΔCt^ method. Three independent biological replicates were performed per treatment.

### Functional Analysis via Virus-Induced Gene Silencing Experiments

Target fragments for silencing (V1 and V2) that had no or low similarity with other genes and thus unlikely to cause off-target effects were selected based on predictions made using si-Fi software (http://www.snowformatics.com/si-fi.html). The plasmids (γ-V1 and γ-V2) utilized for gene silencing were constructed according to the methods described by Holzberg et al. (2002). Plasmids α, β, γ, γ-PDS, γ-V1, and γ-V2 were each linearized. The linearized plasmids were used as templates for *in vitro* transcription using the mMESSAGE mMACHINE^®^ Kit High Yield Capped RNA Transcription Kit (Ambion) according to the manufacturer’s protocol. BSMV VIGS vectors harboring target gene sequences and *Pt* urediospores were co-inoculated into wheat cultivars TcLr19 and Thatcher according to previously described methods (Wang et al., 2020). Plants mock inoculated with sterile water and plants inoculated with a VIGS vector targeting the wheat phytoene desaturase (*TaPDS*) gene were used as negative and positive controls, respectively.

### Detection of Hydrogen Peroxide Accumulation, Enzyme Activity, and Expression of the Marker Genes

H_2_O_2_ accumulation was detected using 3,3-diaminobenzidine (DAB; Coolaber, China) staining as described by Thordal-Christensen et al. (1997) and observed with a Nikon Ti2-LAPP Ti2 Laser Application System (Nikon Corporation, Japan). A minimum of 50 infection sites were examined for every treatment. Superoxide dismutase (SOD) and catalase (CAT) enzyme activities were detected using Total Superoxide Dismutase Assay Kit with NBT (Leagene) and Catalase Assay Kit with Ultraviolet Colorimetry (Leagene), respectively, according to the manufacturers protocol. In addition, the expression levels of marker genes, *TaPR1* (GenBank: HQ848391), *TaPR2* (GenBank: HQ848392), *TaTLP1* (GenBank: KJ764822), *TaSOD* (GenBank: CB307850), and *TaCAT* (GenBank: X94352), were analyzed by qPCR in silenced plants. Each treatment included three independent biological replicates.

### Identification of Transcriptional Activity

To confirm the presence of an activation domain of TaNAC069, a yeast assay system was used as described previously (Fujita et al., 2010). The recombinant plasmids pBD-TaNAC069, pBD-TaNAC069-N (1–417 bp), pBD-TaNAC069-C (418–1065 bp), and pGBKT7 (negative control) were each transformed into yeast strain AH109. The transformed yeast cells were individually streaked on selective dropout/-tryptophan (SD/-Trp) medium and selective dropout/-tryptophan/-histidine/-adenine with X-α-Gal (SD/-Trp/-His/-Ade/X) medium and then incubated at 30°C for 3 days. The recombinant vectors pBD-TaNAC069-N and pAD-TaNAC069 were constructed and co-transformed into yeast strain Y2HGold to verify the self-interaction of TaNAC069. Each yeast strain was incubated on SD/-Trp and SD medium in the absence of tryptophan, leucine, histidine, and adenine with X-α-Gal (SD/Trp-/Leu-/His-/Ade-/X), respectively.

For GUS staining, the *TaNAC069* promoter fragment was inserted into the pBI121 vector digested with *Hind*III and *Xba*I, using the primer pair pBI121-pTaNAC069-F/pBI121-pTaNAC069-R (Supplementary Table 1). The recombinant plasmid, pBI121-pTaNAC069, was transformed into *Agrobacterium tumefaciens* strain *GV3101* by electroporation and then agroinfiltrated into *Nicotiana benthamiana* plants (grown for 45 days) (Yang et al., 2019). At 72 hpi, inoculated leaves were collected and immediately treated with GUS staining solution. Under the naked eye or microscope, the small blue dots on the white background indicate GUS expression sites.

To generate the GFP constructs, the *TaNAC069* promoter fragment was recombined into the vector pCamA-GFP digested with *Hind*III and *Kpn*I, using the primer pair pCamA-pTaNAC069-F/pCamA-pTaNAC069-R (Supplementary Table 1). The recombinant plasmid pCamA-pTaNAC069-GFP was used for *Agrobacterium-*mediated transformation.

### Yeast One-Hybrid and Yeast Two-Hybrid Assays

For yeast one-hybrid assays, the recombinant yeast strain Y1HGold (pAbAi-pTaNAC069) was identified via PCR using Matchmaker Insert Check PCR Mix 1 (Clontech, Palo Alto, CA, United States) as described by Yang et al. (2019). Yeast library plasmids (10 μg) were transformed into the recombinant yeast strain Y1HGold (pAbAi-pTaNAC069). Healthy single clones were selected for colony PCR using Matchmaker Insert Check PCR Mix 2 (Clontech, Palo Alto, CA, United States) to obtain the inserted fragment of the cDNA library. The amplified products (1–2 kb) were purified by agarose gel electrophoresis and sequenced at Sangon Biotech, Shanghai, China, using the T7 promoter sequence as primer. The sequencing results were analyzed using NCBI (http://blast.ncbi.nlm.nih.gov/Blast.cgi).

For yeast two-hybrid assays, the bait vector pGBKT7-TaNAC069-N was transformed into Y2HGold yeast strain using the LiAc (Clontech, Palo Alto, CA, United States) and confirmed by toxicity and self-activation. Screening of interacting targets of TaNAC069 was done using the Matchmaker Gold Yeast Two-Hybrid (Y2H) System (Clontech, Palo Alto, CA, United States). Positive colonies were selected for colony PCR using Matchmaker Insert Check PCR Mix 2 (Clontech, Palo Alto, CA, United States) and sequenced at Sangon Biotech, Shanghai, China, using the T7 primer.

### Data Availability Statement

The data presented in this study can be found in online repositories. The data have been deposited in NCBI. The SRA accession number is SRR13254555. The TSA accession number is GIXT00000000.

## Results

### *TaNAC069* Was Obtained Based on RNA-Seq Data

**FIGURE 1:**
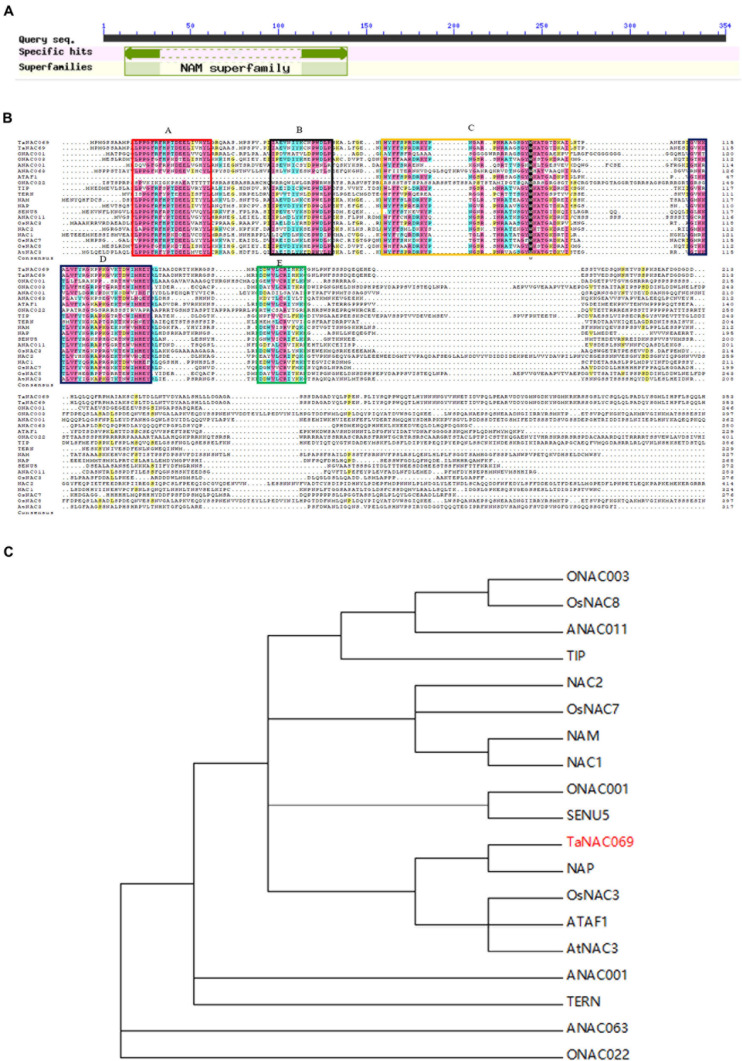
Analysis of *TaNAC069* gene. **(A)** Structural domains of TaNAC069. **(B)** Multiple alignments of *TaNAC069* and homologous genes. **(C)** Phylogenetic analysis of NACs. NAC subdomains (A–E) are conserved and underlined. Identical and similar residues are shaded in different colors, respectively. The unrooted phylogenetic tree of NAC domains was depicted using the CLUSTAL X program and was constructed using the neighbor-joining method. ANAC001, *Arabidopsis thaliana* NAC001 (AT1G01010); NAC1, *A. thaliana* NAC1 (AF198054); NAC2, *A. thaliana* NAC2 highly expressed in root meristem (AF201456); AtNAC3, *A. thaliana* NAC3 (AB049070); ANAC011, *A. thaliana* NAC011 (AT1G32510); ANAC063, *A. thaliana* NAC063 (AT3G55210); ATAF1, *Arabidopsis* transcription activation factor 1 (X74755); TIP, *A. thaliana* TIP interacts with turnip crinkle virus capsid protein (AF281062); ONAC001, *Oryza sativa* NAC001 (AK060509); ONAC003, *O. sativa* NAC003 (AK061716); OsNAC3, *O. sativa* NAC3 (AB028182); *OsNAC7*, *O. sativa NAC7* (AB028186); OsNAC8, *O. sativa* NAC8 (AB028187); ONAC022, *O. sativa* NAC022 (AK107090); TERN, tobacco elicitor-responsive (AB021178); NAM, *Petunia hybrida* no apical meristem (X92204); NAP, NAC-like, activated by AP3/PI (AJ222713); SENU5, *Lycopersicon esculentum* SENU5 (Z75524).

A 1259-bp fragment was identified and cloned based on RNA-seq data via RT-PCR (Supplementary Figure 1); sequence analysis showed that it belongs to the NAC transcription factor family, as expected. The open reading frame (ORF) finder showed that the corresponding gene comprises a 1065-bp complete ORF encoding 354 amino acids, including the N-terminal 127 (13–139) amino acid residue NAM domain and the C-terminal highly variable transcriptional regulatory region (Figure 1A). A BLASTp search showed that the amino acid sequence was highly homologous with published sequences of wheat (*T. aestivum*) TaNAC69 (AAU08785.1), with homology of 98.87%; it is named *TaNAC069*. BLAST programs in the Chinese spring wheat database (http://plants.ensembl.org/index.html) show that *TaNAC069* is highly similar to its homologs located on chromosomes 5D (TraesCS5D02G148800.1) and 5B (TraesCS5B02G142100.1). Further analysis confirmed its homology with typical NAC transcription factors, such as *Arabidopsis thaliana* ATAF1 (X74755) (36.6%), *A. thaliana* TIP (AF281062) (46.91%), *Oryza sativa* OsNAC3 (AB028182) (54.50%), *Petunia hybrida* NAM (X92204) (54.49%), NAC-like NAP (AJ222713) (56%), and *Lycopersicon esculentum* SENU5 (Z75524) (54.22%) (Figure 1B). *TaNAC069* contains all A, B, C, D, and E conserved subdomains, which were confirmed by alignment with typical NAC transcription factors representing different NAC phylogenetic subgroups (Ooka et al., 2003). It was found to belong to the NAP subfamily *via* phylogenetic analysis of *TaNAC069* (Figures 1B,C).

### *TaNAC069* Expression Was Significantly Induced in TcLr19 Plants Upon *Pt* Infection

**FIGURE 2:**
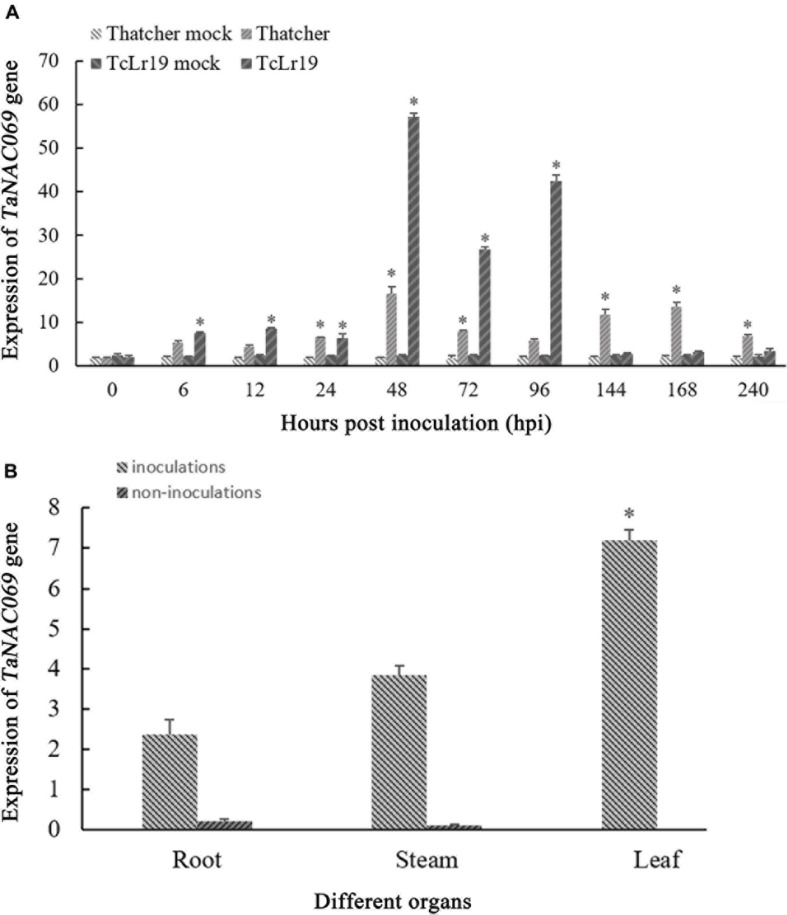
Analysis of *TaNAC069* gene expression. **(A)** Expression profiles of the *TaNAC069* gene in wheat infected with *Pt*. **(B)** Expression profile of the *TaNAC069* gene in different organs. The *y*-axis indicates the amounts of gene transcripts normalized to the *GAPDH* gene and expressed relative to that of mock-inoculated control plants treated with 0.1% (v/v) ethanol solution. The *x*-axis indicates different sampling. The reference gene is *GAPDH*. The assays for each treatment consisted of three biological replicates. Data are means ± standard errors of three independent experiments. Differences between time-course points were assessed using SSPS. ^∗^*p* < 0.05, *n* = 3.

qPCR was used to investigate the expression of *TaNAC069* in response to *Pt* infection. The expression level of *TaNAC069* during incompatible interactions increased from 0 to 24 hpi, peaking at 48 hpi (about 23.8 times higher than the 0 hpi control), and then decreased at 72 hpi. A second expression peak observed at 96 hpi was lower than the first but 20.1 times higher than the control (Figure 2A). During compatible interactions, *TaNAC069* expression was up-regulated compared with the non-inoculated control at most time points (Figure 2A). It exhibited expression peaks at 48 hpi (8.9 times higher than the control) and 168 hpi (7.3 times higher than the control) and then declined. Overall, the expression level of *TaNAC069* was higher during incompatible interactions than compatible ones at most time points, indicating that *TaNAC069* expression was induced by *Pt* infection.

Additionally, the expression level of *TaNAC069* was low in non-inoculated roots and stems and exhibited almost no expression in wheat leaves. After inoculation with *Pt* race PHNT, higher expression levels of *TaNAC069* were observed in leaves and stems than in roots, with the expression in leaves being 3.0 times higher than in roots and 1.9 times than in stems (Figure 2B). *TaNAC069* expression showed a significant variation in different plant tissues before and after inoculation with PHNT, indicating that expression is tissue specific and induced by the leaf rust pathogen.

### Induction of *TaNAC069* by Chemical Reagents

**FIGURE 3:**
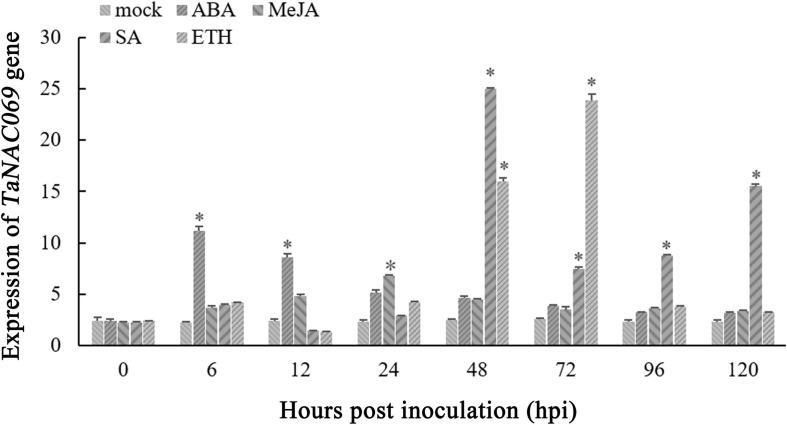
Expression profiles of the *TaNAC069* gene in response to different chemical inducers. The *y*-axis indicates the amounts of gene transcripts normalized to the *GAPDH* gene and expressed relative to mock-inoculated control plants treated with 0.1% (v/v) ethanol solution. The *x*-axis indicates different sampling timepoints. The reference gene is *GAPDH*. The assays for each treatment consisted of three biological replicates. Data are means ± standard errors of three independent experiments. Differences between time-course sampling points were assessed using SSPS. ^∗^*p* < 0.05, *n* = 3.

To better understand the signal pathways of *TaNAC069*, the expression profiles of *TaNAC069* induced by ABA, SA, ETH, and MeJA were analyzed. The expression level of *TaNAC069* increased from 0 to 6 h after ABA treatment and was markedly high at 6 hpt, being nearly 4.7 times higher than that of the control, followed by a slight decrease. During SA treatment, the *TaNAC069* expression levels peaked at 48 hpt, which was nearly 11.2 times higher than those of the control, followed by a sharp decrease from 48 to 96 hpt and a subsequent increase at 96 hpt, and peaked again at 120 hpt. The expression level of *TaNAC069* was significantly increased, peaking at 72 hpt, after ETH treatment, which was nearly 10.1 times higher than in the control, then followed by a decrease (Figure 3). MeJA treatment had no obvious effect on *TaNAC069* expression. The above-mentioned results show that *TaNAC069* expression was induced by ABA and SA, experiencing an earlier induction by ABA together with ETH; however, it was not significantly induced by MeJA.

### *TaNAC069* Knockdown Decreased the Resistance of Wheat Against *Pt*

**FIGURE 4:**
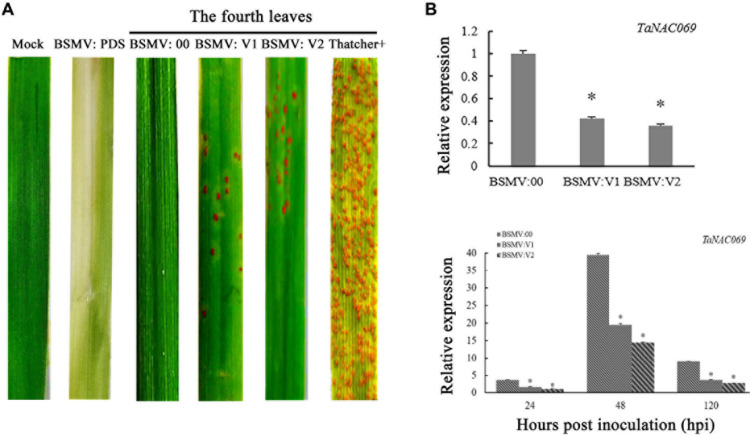
Functional analysis of the *TaNAC069* gene in response to leaf rust infection using virus-induced gene silencing. **(A)** Disease symptoms of *TaNAC069* gene-knockdown wheat leaves 14 days after inoculation with *Pt* pathotype PHNT. **(B)** Relative expression of wheat *TaNAC069* in gene-knockdown wheat leaves after inoculation with PHNT. Mock, TcLr19 wheat leaves without barley stripe mosaic virus (BSMV) and PHNT inoculation; BSMV:00, TcLr19 after BSMV and PHNT inoculation; Tc+, Thatcher after PHNT inoculation. The reference gene is *GAPDH*. The assays for each treatment and phenotype combination consisted of at least three biological replicates (BSMV:00 was used as the control). Data are means ± standard errors of three independent experiments. Differences between time-course sampling points were assessed using SSPS. ^∗^*p* < 0.05, *n* = 3.

To confirm the function of *TaNAC069* in wheat resistance to *Pt*, the gene was knocked down using the VIGS system. Knockdown of the *PDS* gene in plants produces a visible photo-bleaching phenotype that can be used as a positive control to confirm the proper functioning of the VIGS system. Mock (buffer inoculated without BSMV) and BSMV:00 (empty vector)-inoculated plants displayed an immune phenotype (scale 0) and no other obvious defects at 14 days post-inoculation with *Pt* pathotype PHNT compared to Thatcher (scale 4), which indicated that virus inoculation did not affect the wheat–*Pt* incompatible interaction. BSMV:V1 and BSMV:V2 inoculated with PHNT exhibited more *Pt* uredinia (scale 3) compared with the mock and BSMV:00 treatments. Thus, knocking down the expression of the *TaNAC069* gene reduced the resistance of wheat against PHNT (Figure 4A).

qPCR was used to determine the expression level of *TaNAC069* in each treatment. Without inoculation, the expression level of *TaNAC069* in BSMV:V1 and BSMV:V2 was reduced significantly, and the silencing efficiency was more than 50% compared to BSMV:00 (Figure 4B). After inoculation with PHNT, compared to BSMV:00, the expression of *TaNAC069* was significantly reduced to 56%, 51%, and 60% in BSMV:V1 at 24, 48, and 120 h, respectively, and for the BSMV:V2 treatment expression, it was reduced to 69%, 64%, and 70% at 24, 48, and 120 h, respectively (Figure 4B).

**FIGURE 5:**
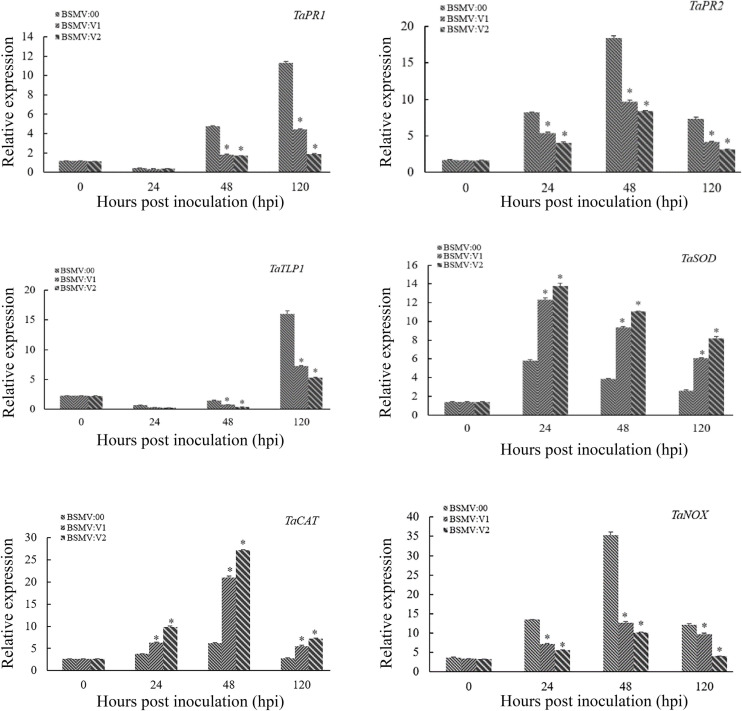
Relative transcript levels of *TaPR1*, *TaPR2*, *TaTLP1*, *TaSOD*, *TaCAT*, and *TaNOX* in *TaNAC069*-knockdown plants inoculated with PHNT. The *y*-axis indicates the amounts of gene transcripts normalized to the *GAPDH* gene and expressed relative to that of mock-inoculated control plants treated with 0.1% (v/v) ethanol solution. The *x*-axis indicates different sampling timepoints. The reference gene is *GAPDH*. The assays for each gene consisted of at least three biological replicates. Data are means ± standard errors of three independent experiments. Differences between time-course sampling points were assessed using SSPS. ^∗^*p* < 0.05, *n* = 3.

To determine whether TaNAC069 activates or represses the expression of downstream defense-related genes, several pathogenesis-related (PR) genes and reactive oxygen species (ROS)-related genes were selected and analyzed via qPCR. The expression of *TaPR1*, *TaPR2*, *TaTLP1*, and ROS synthesis-related genes (*TaNOX*) was significantly lower in the *TaNAC069*-knockdown plants compared with the control, and the expression of ROS clearance-related genes (*TaCAT* and *TaSOD*) was significantly higher (Figure 5).

**FIGURE 6:**
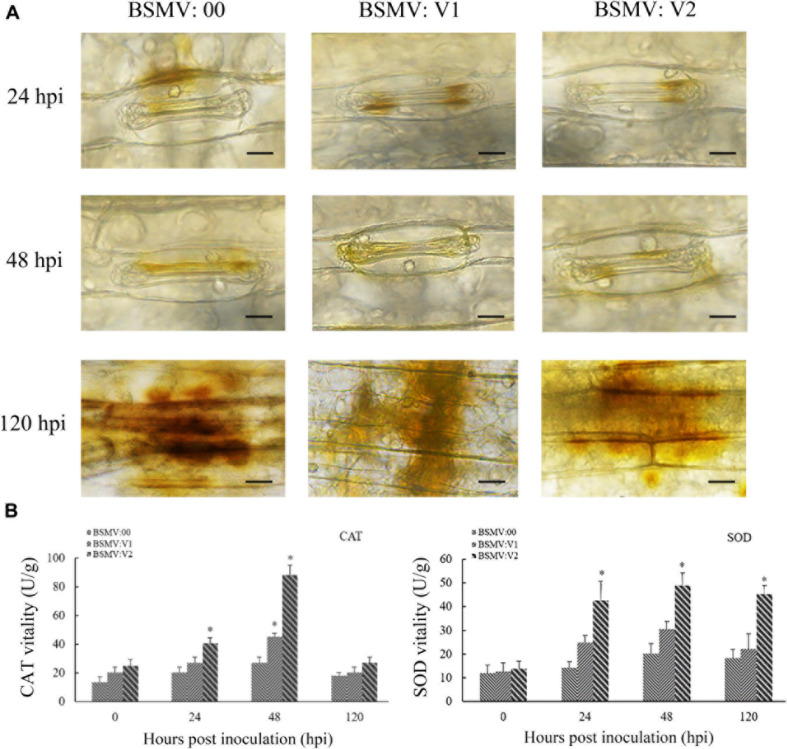
Determination of leaf H_2_O_2_ concentration by 3,3-diaminobenzidine (DAB) staining. **(A)** H_2_O_2_ accumulation in *TaNAC069*-knockdown wheat plants was detected by staining with DAB and viewed under differential interference contrast optics. Scale bar, 50 μm. **(B)** Superoxide dismutase (SOD) and catalase (CAT) activity in *TaNAC069*-knockdown wheat plants. The *y*-axis indicates the amount of SOD and CAT activities (each treatment and phenotype combination consisted of at least three biological replicates). The *x*-axis indicates different sampling timepoints. Data are means ± standard errors of three independent experiments. Differences between time-course sampling points were assessed using SSPS. ^∗^*p* < 0.05, *n* = 3 (BSMV:00 used as the control).

H_2_O_2_ accumulation by DAB staining and related enzyme activity (CAT and SOD) were measured during the wheat–*Pt* interaction. As shown in Figure 6, H_2_O_2_ accumulation was significantly reduced and the activities of SOD and CAT enzymes were significantly increased in *TaNAC069*-knockdown plants compared with the control. These results indicate that the *TaNAC069* gene plays a positive regulatory role in wheat resistance to *Pt*.

### Identification of TaNAC069 and pTaNAC069 Activity

**FIGURE 7:**
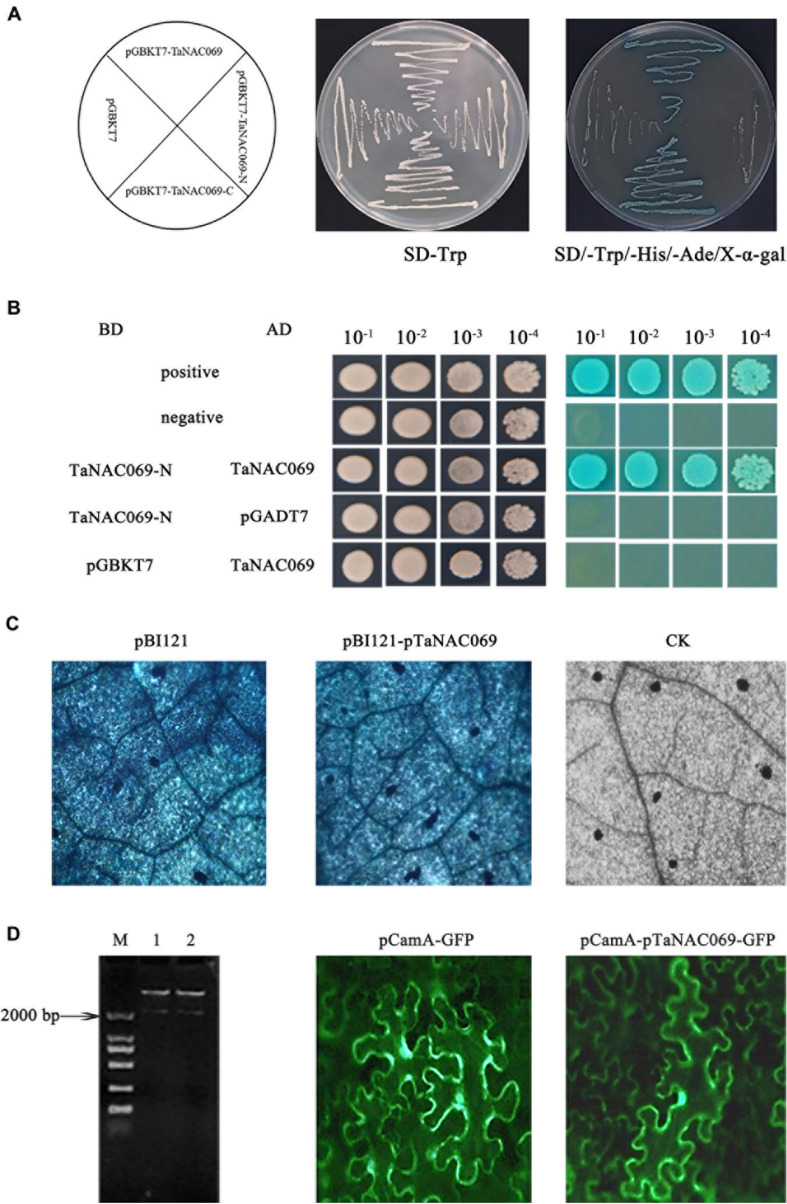
Identified activity of TaNAC069 and pTaNAC069. **(A)** Transcription activation activity of the TaNAC069. **(B)** Homocomplex formation of the TaNAC069 in yeast. **(C)** GUS histochemical staining directed by the pTaNAC069 sequences in tobacco. pBI121, GUS histochemical staining results of tobacco leaves transformed by vector pBI121; pBI121-pTaNAC069, GUS histochemical staining results of tobacco leaves transformed by pBI121-pTaNAC069; CK, GUS histochemical staining results of tobacco. Scale bars are 50 μM. **(D)** GFP expressed by the pTaNAC069 sequences in tobacco. M, Marker 2000; 1–2, restrictive digestion of the pCamA-pTaNAC069-GFP plasmid; pCamA-GFP, results of tobacco leaves transformed by vector pCamA; pCamA-pTaNAC069-GFP, results of tobacco leaves transformed by pCamA-pTaNAC069-GFP. Scale bars are 50 μM.

In order to examine the transcriptional activity and self-interaction of TaNAC069, the yeast-transformed system was used. All transformed yeast strains grew well on SD/-Trp medium, indicating that these vectors were successfully transformed into yeast cells. Yeast cells containing pBD-TaNAC069 or TaNAC069-C grew well and turned blue on SD/-Trp/-His/-Ade/X-α-Gal, whereas cells harboring pGBKT7 or pBD-TaNAC069-N did not (Figure 7A). Yeast cells containing pBD-TaNAC069-N and pAD-TaNAC069 in combination and the positive controls grew well and turned blue on SD/-Trp/-Leu/-His/-Ade/X. In contrast, replacement of either the pBD-TaNAC069-N or the pAD-TaNAC069 with vectors expressing only the AD or BD yeast proteins resulted in no production of blue coloration (Figure 7B). These results suggest that TaNAC069 exhibits transactivation activity, that the C-terminus can be attributed to transcriptional activation, and that TaNAC069 forms NAC protein homomeric dimers which possess unique biological functions.

To further understand how *TaNAC069* is regulated, the promoter with a length of 2,103 bp (upstream of the translational initiation codon ATG) was cloned by RT-PCR (Supplementary Figure 2). The sequence shared 98% identity compared with the wheat genome; the sequence was given the label *pTaNAC069*. Analysis of the *pTaNAC069* sequence using PlantCARE software and PLACE database revealed that two classical promoter *cis*-acting elements, TATA-box (-1,977, -385, and -201 bp upstream from the transcription start site) and CAAT-box (-1435, -1136, and -878 bp upstream from the transcription start site), were present. *cis*-Acting motifs of the *pTaNAC069* sequence were categorized into five major groups as follows: conserved motifs, abiotic stress, pathogens, phytohormones, and tissue-specific expression-responsive *cis*-regulatory motifs (Table 1 and Supplementary Figure 3) according to Tiwari et al. (2019). The CaMV35S promoter in the pBI121 vector was replaced by *pTaNAC069* using the seamless cloning method to form the recombinant plant expression vector pBI121-*pTaNAC069* (Supplementary Figure 4) to verify the promoter activity of *pTaNAC069*. As shown in Figure 7C, the results of GUS staining revealed a strong blue signal in *N. benthamiana* leaves injected with pBI121 and pBI121-*pTaNAC069* that was not likewise observed for the control (normal leaves). Additionally, the promoter region of pCamA-GFP was replaced by *pTaNAC069*. As shown in Figure 7, GFP fluorescence was uniformly distributed throughout the cells of leaves injected with pCamA-GFP and pCamA-pTaNAC069-GFP, respectively, indicating the functional promoter activity of pTaNAC069.

**TABLE 1:** *Cis*-acting elements identified on the -2103 bp (upstream to ATG) putative promoter region of *TaNAC069* using PlantCARE and PLACE database.

Category	Site name (No.)	Sequence	Function	Position	Organism
Conserved motifs	CAAT-box (3)	CCAAT	Common *cis*-acting element in promoter and enhancer regions	−1435	*Arabidopsis thaliana*
		CCAAT		−1136	*A. thaliana*
		CAAT		−878	*Nicotiana glutinosa*
	TATA-box (3)	TATA	Core promoter element around -30 of transcription start	−1977	*A. thaliana*
		ATATAA		−385	*Brassica oleracea*
		TATAAA		−201	*A. thaliana*
Abiotic stress responsive	Box 4 (1)	ATTAAT	Involved in light responsiveness	−1069	*Petroselinum crispum*
	AE-box (1)	AGAAACAA	Part of a module for light response	−534	*A. thaliana*
	G-box (5)	CACGTG	Involved in light responsiveness	−1194	*A. thaliana*
		CACGAC		−622	*Zea mays*
		CACGTG		−1055	*A. thaliana*
		CACGAC		−646	*Z. mays*
		TACGTG		−310	*A. thaliana*
	CCAAT-box (1)	CAACGG	MYBHv1 binding site	−1996	*Hordeum vulgare*
	DRE1 (2)	ACCGAG	dehydration responsive element	−1642	*Z. mays*
		ACCGAG		−1676	*Z. mays*
Pathogen responsive	W box (1)	TTGACC	Fungal elicitor responsive element	−1498	*A. thaliana*
Phytohormone responsive	ABRE (5)	ACGTG	In the abscisic acid responsiveness	−309	*A. thaliana*
		ACGTG		−506	*A. thaliana*
		ACGTG		−618	*A. thaliana*
		ACGTG		−1193	*A. thaliana*
		ACGTG		−1056	*A. thaliana*
	GARE-motif (1)	TCTGTTG	Gibberellin-responsive element	−1259	*B. oleracea*
	TGA-box (1)	TGACGTAA	Part of an auxin-responsive element	−303	*Glycine max*
	TGACG-motif (1)	TGACG	Involved in the MeJA-responsiveness	−303	*H. vulgare*
Tissue specific expression	A-box (1)	CCGTCC	*cis*-Acting regulatory element	−740	*P. crispum*

### Upstream Transcription Factors and Interacting Targets of TaNAC069 Were Determined

In order to determine the potential interacting targets of TaNAC069, the bait vector pAbAi-*pTaNAC069* was successfully constructed and transformed into Y1Hgold (Supplementary Figures 5A,B). As shown in Supplementary Figure 5C, the minimum concentrations of AbA needed to suppress the basal expression of *pTaNAC069* and *p53* bait strains were 200 and 100 ng/ml, respectively. In total, 410 positive colonies were identified and analyzed by yeast one-hybrid assays. A total of 45 positive colonies exhibiting PCR products with bright bands and over 1000-bp-long fragments were sequenced by Sangon Biotech, Shanghai, China (Supplementary Figure 5D). Moreover, 11 interaction targets binding to the *TaNAC069* promoter region were selected and analyzed, including glutathione peroxidase (AJE63414.1), eukaryotic elongation factor *eEF1A* (AQU14666.1), CBS domain-containing protein CBSX3 (XP_020173473.1), cold acclimation protein WCOR410c (AAB18202.1), peptidyl-prolyl *cis*–*trans* isomerase (4E1Q_A), chlorophyll A–B binding protein (IPR023329), lipid transfer protein precursor (AAK20395.1), GDT1-like protein 5, and unnamed protein product (VAI25758.1) (XP_020177476.1) (Table 2).

**TABLE 2:** Screening results of *TaNAC069* promotor transcriptional factors.

Number	Fragment length (bp)	Blastx	NCBI number	Biological annotation	Species
1, 247	903/867	Blastx	AQU14666.1	Eukaryotic elongation factor eEF1A	*Triticum aestivum*
208	780/777	Blastx	AAB18202.1	Cold acclimation protein WCOR410c	*T. aestivum*
262	618/615	Blastx	XP_020173473.1	CBS domain-containing protein CBSX3, mitochondrial	*Aegilops tauschii*
203	507/504	Blastx	AJE63414.1	Glutathione peroxidase, GPX	*T. aestivum*
206	842/512	Blastx	4E1Q_A	Peptidyl-prolyl *cis*–*trans* isomerase	*T. aestivum*
7	1032/780	Blastx	IPR023329	Chlorophyll A–B binding protein	*T. aestivum*
268	444/255	Blastx	AAK20395.1	Lipid transfer protein precursor	*T. aestivum*
271	411/387	Blastx	VAI25758.1	Unnamed protein product	*T. turgidum*
257	451/435	Blastx	XP_020184078.1	Protein CURVATURE THYLAKOID 1B, chloroplastic-like isoform X2	*A. tauschii*
209	699/696	Blastx	XP_020177476.1	GDT1-like protein 5 isoform X2	*A. tauschii*

The bait vector pGBKT7-TaNAC069-N was successfully constructed and confirmed to have no toxicity or self-activation in the yeast strain Y2HGold (Supplementary Figure 6). In total, 450 positive colonies were identified and analyzed via the bait vector pGBKT7-TaNAC069-N and yeast library mating (Supplementary Figure 6C). A total of 60 positive colonies, exhibiting PCR products with bright bands and over 750–2000-bp-long fragments, were sequenced. The sequencing results were selected and analyzed via BLAST. A total of 55 target protein sequences were identified in wheat (Table 3), including seven papain family cysteine protease (XP_020166016.1), three hypersensitive induced reaction protein 1 (AFD54041.1), two elongation factors (EMS67172.1, AQU14666.1), three drought-induced 19 protein (Di19) (AIU99984.1), seven protein EARLY RESPONSIVE TO DEHYDRATION 15-like (ERD15) (XP_020186168.1), one salicylic acid-binding protein 2-like (XP_020164120.1), one CAT (AIZ77475.1), one E3 ubiquitin-protein ligase UPL6 (XP_020165537.1), one probable indole-3-acetic acid-amido synthetase (XP_020153870.1), and 1,3-1,4-β-D-glucanase-like (XP_020192465.1) in addition to others. Five target protein sequences were identified in *Pt* (Table 4), including hypothetical protein PTTG_06575, hypothetical protein PTTG_01234, NADH dehydrogenase, asparaginyl-tRNA synthetase, and 5-methyltetra hydropteroyl triglutamate-homocysteine.

**TABLE 3:** Screening results of the *TaNAC069* target in the host.

No.	Fragment length (bp)	NCBI number	Biological annotation	Species	Number
32,143,135,99,129,117,62	866/504	XP_020166016.1	Papain family cysteine protease	*A. tauschii*	7
38,41,147	669/660	AFD54041.1	Hypersensitive induced reaction protein 1	*T. aestivum*	3
40	651/639	EMS67172.1	Elongation factor 2	*T. urartu*	1
01	657/654	AQU14666.1	Eukaryotic elongation factor eEF1A	*T. aestivum*	1
59,63,34	705/666	AIU99984.1	Drought-induced 19 protein	*T. aestivum*	3
37,5,15,83,140,54,107	988/255	XP_020186168.1	Protein EARLY RESPONSIVE TO DEHYDRATION 15-like, ERD15	*A. tauschii*	7
6	765/729	AIZ77475.1	Catalase, partial	*T. aestivum*	1
03	2574/2571	XP_020165537.1	E3 ubiquitin-protein ligase UPL6 isoform X1	*A. tauschii*	1
68	1098/864	XP_020153870.1	Probable indole-3-acetic acid-amido synthetase GH3.8	*A. tauschii*	1
56	861/567	XP_020192465.1	Endo-1,3;1,4-β-D-glucanase-like	*A. tauschii*	1
27	919/399	XP_020164120.1	Salicylic acid-binding protein 2-like	*A. tauschii*	1

**TABLE 4:** Screening results of the *TaNAC069* target in *Pt*.

No.	Biological annotation	Species	Signal peptide	Cys number	Amino acid	Subcellular localization
43	Hypothetical protein PTTG_06575	*Puccinia triticina 1-1 BBBD Race 1*	No	2	142	Endoplasmic reticulum
42	NADH dehydrogenase (quinone), G subunit	*Pu. triticina 1-1 BBBD Race 1*	No	15	238	Nuclear
2-2	Hypothetical protein PTTG_01234	*Pu. triticina 1-1 BBBD Race 1*	No	5	289	Nuclear
78	5-Methyltetra hydropteroyl triglutamate-homocysteine methyltransferase	*Pu. triticina 1-1 BBBD Race 1*	No	2	192	Endoplasmic reticulum
407	Asparaginyl-tRNA synthetase	*Pu. triticina 1-1 BBBD Race 1*	No	3	186	Cytoplasmic

### Confirmation of TaNAC069 Interaction With *TaCP5* by Y2H Assay

**FIGURE 8:**
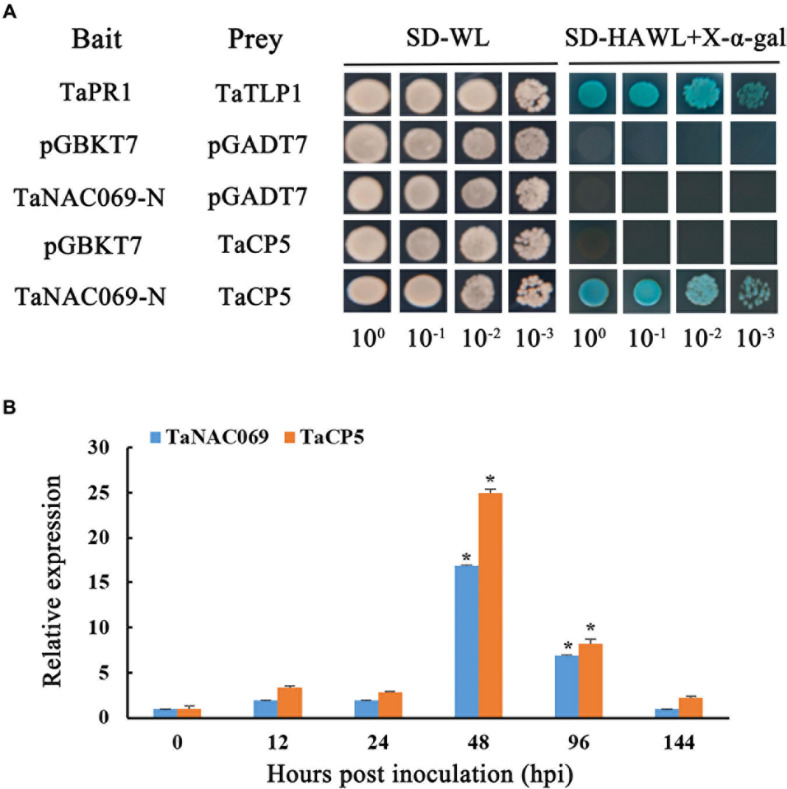
Interaction between TaNAC069 and TaCP5. **(A)** Yeast two-hybrid analysis of the interaction between TaNAC069 and TaCP5 in yeast. Yeasts expressing the indicated combinations of bait and prey proteins were spotted onto synthetic dropout medium lacking leucine and tryptophan (SD-WL) and SD medium lacking leucine, tryptophan, histidine, and adenine supplemented with X-α-Gal and Aureobasidin A (SD-HAWL + X-α-Gal). Only yeast strains co-expressing TaNAC069 and TaCP5 grew on SD-HAWL plates and developed blue coloration, indicative of an interaction between both proteins. TaPR1 and TaTLP1 are the positive controls; TaNAC069-N and pGADT7, TaCP5 and pGBKT7, and pGADT7 and pGBKT7 were used as negative controls. **(B)** Gene expression profiles of TaNAC069 and TaCP5 in wheat infected with *Pt* at different timepoints post-inoculation. The *y*-axis indicates the amounts of gene transcripts normalized to the *GAPDH* gene and expressed relative to plants at 0 hpi. The *x*-axis indicates different sampling timepoints. The reference gene is *GAPDH*. The assays for each treatment included three biological replicates. Data are means ± standard errors of three independent experiments. Differences between time-course sampling points were assessed using SSPS. *p < 0.05, *n* = 3.

Zhang et al. (2019) reported that *TaCP3*, a cysteine protease family gene and a type of papain protease, plays a role in drought stress, so the papain family cysteine protease was selected for further investigation. BLASTp analysis showed that the papain family cysteine protease, labeled as screened by TaNAC069, shared 100% homology with published sequences (TraesCS5B02G208100.1) and was given the label *TaCP5*. To test if TaNAC069 can interact with TaCP5, pGADT7-TaCP5 plasmids were co-transformed into the yeast strain Y2HGold and cultured on the selection medium SD/-Trp/-Leu/-His/-Ade and X-α-gal together with construct pBD-TaNAC069-N. The results showed that the yeast transformant co-expressing the pBD-TaNAC069-N and pGADT7-TaCP5 plasmids produced blue coloration (Figure 8A, rows 5) similar to the positive control (Figure 8A, rows 1). In contrast, replacement of either the pBD-TaNAC069-N prey or the pGADT7-TaCP5 bait with vectors expressing either the AD or BD yeast proteins produced no blue coloration (rows 2–4).

To further explore their potential molecular functions in regulation relationship between wheat and *Pt*, the co-expression patterns of *TaCP5* were tested in the same manner as *TaNAC069*. We found that the expression level of the *TaNAC069* gene in TcLr19 plants at different sampling time points after inoculation with *Pt* were similar to *TaCP5* (Figure 8B), suggesting that the expression of both *TaNAC069* and *TaCP5* is induced by leaf rust infection and shares similar expression profiles, supporting the hypothesis that TaNAC069 and TaCP5 are likely to interact with each other.

## Discussion

Many studies have shown that NAC transcription factors have a variety of biological functions and play important roles in plant resistance to disease. Xue et al. (2006) isolated 14 *TaNAC69* clones which were grouped into three highly homologous *TaNAC69* gene categories (*TaNAC69-1*, *TaNAC69-2*, *TaNAC69-3*). *TaNAC69* genes are likely to have some homologous genes due to the allopolyploid nature of hexaploid wheat. By analyzing Affymetrix expression data, *TaNAC69* was found to be up-regulated in rust-infected wheat leaves and involved in regulating wheat adaptation to drought stress (Xue et al., 2011). The expression of *TaNAC4* isolated from wheat (*T. aestivum* L.) leaves was significantly induced by *P. striiformis* f. sp. *tritici* (*Pst*) (Xia et al., 2010), and the expression of *BnNAC56* isolated from oilseed rape (*Brassica napus*) was rapidly and significantly induced by *Sclerotinia sclerotiorum* (Chen et al., 2017). In this study, *TaNAC069*, which is highly homologous to *TaNAC69*, was cloned and induced by *Pt* infection. To assess the role of TaNAC069 in wheat–*Pt* interactions, the *TaNAC069* gene was knocked down using a BSMV–VIGS system, which has been successfully used to identify gene function in plants (Yang et al., 2018). The *TaNAC069*-silenced wheat plants exhibited reduced resistance against *Pt*, suggesting that *TaNAC069* plays a positive role in wheat resistance against *Pt.*

NAC transcription factors could activate defense-related genes and enhance plant disease resistance against various stress conditions. For example, *MaNAC5* interacts with *MaWRKY1/2* to regulate the expression of specific PR genes in banana (*Musa acuminata*) against the fungus *Colletotrichum musae* (Shan et al., 2016). *OsNAC4*, a transcription factor in rice, positively regulates the HR (Kaneda et al., 2009). H_2_O_2_ accumulation is observed in a variety of defense responses, including those induced by wounding, insect feeding, and pathogen infection (Orozco-Cardenas and Ryan, 1999). H_2_O_2_ is a reactive oxygen species, which is relatively stable and participates in the HR. Balazadeh et al. (2010) reported that some NACs are involved in H_2_O_2_-mediated signaling in *Arabidopsis*. *TaNAC2* is likely to participate in resistance responses through the generation of H_2_O_2_ during wheat–*Pst* compatible interactions (Zhang et al., 2018). In the present study, we found that the expression of PR genes, ROS-related genes, and H_2_O_2_ accumulation were significantly induced in *TaNAC069*-knockdown plants compared with controls. Based on these results, we believe that *TaNAC069* enhances wheat resistance to the leaf rust pathogen through transcriptional regulation of specific downstream disease-related resistance genes.

Yeast one-hybrid assay is widely recognized as a useful technique for detecting physical interactions between DNA and DNA-binding proteins. In this study, several potential transcription factors were successfully screened by yeast one-hybrid assay, including *WCOR410C*, *CBSX3*, and *eEF1A*. *WCOR410C* is a member of the dehydrin (dehydration) multi-protein family and encodes a peripheral protein that is responsive to numerous abiotic and ABA stresses (Kiani et al., 2020). Dehydration-responsive element-binding proteins are a large family of transcription factors that induce the expression of a large number of functional genes responsible for stress endurance in plants (Agarwal et al., 2006). The dehydration-responsive element (DRE), a *cis*-acting element of the promoter regions, was identified (Shinozaki and Yamaguchi-Shinozaki, 2007). ABRE (ABA-responsive element) and DRE1 are present in the *TaNAC069* gene promoter region. CBSX, a protein having only one pair of CBS domains without any other protein domains, binds to specific signal molecules to regulate the activity of various downstream partner proteins. In rice, *OsCBSX3* was identified and up-regulated significantly by the inoculation of *Magnaporthe oryzae* and exogenous application of SA and MeJA (Mou et al., 2015). The TGACG motif of *pTaNAC069* is involved in MeJA responsiveness, and *TaNAC069* was induced by SA. Further experiments, such as Y2H and the electrophoretic mobility shift assay, are needed to determine whether WCOR410C is involved in the transcriptional regulation of *pTaNAC069*, and CBSX3 may regulate the expression of the *TaNAC069* gene by binding to MeJA and SA.

Several potential target proteins were screened by yeast two-hybrid assay, including the salicylic acid-binding protein 2-like, papain family cysteine protease, Di19, and *eEF1A* proteins. *SABP2* activates host SA-mediated defense responses to biotic stress (Park et al., 2007). Sweet potato papain-like cysteine protease, *SPCP2*, is a functional senescence-associated gene (Chen et al., 2010). The expression of *Brassica oleracea* BoCP4 (papain-like cysteine proteases) and *A. thaliana* RD19 (a dehydration-responsive cysteine protease) was dehydration responsive and repressed by water and sucrose (Coupe et al., 2003). In our study, TaNAC069 was found to interact with TaCP5 as confirmed by Y2H. Further experiments such as Y2H, bimolecular fluorescence complementation, and co-immunoprecipitation are needed to determine whether these potential target proteins are involved in interactions with TaNAC069.

Taken together, *TaNAC069* enhances wheat resistance to *Pt* by activating *PR* genes or inhibiting ROS clearance-related genes. Our findings will broaden our understanding of the potentially versatile functions of NACs and provide valuable insight into the molecular mechanisms underlying plant immunity. Further experiments using stable genetic transformation of the *TaNAC069* gene for reliable functional characterization are being carried out, and more than 10 T_0_ lines were obtained to further confirm the function of *TaNAC069*.

## Data Availability Statement

The datasets presented in this study can be found in online repositories. The names of the repository/repositories and accession number(s) can be found below: The data presented in this study can be found in online repositories. The data has deposited in NCBI. The SRA accession number is SRR13254555. The TSA accession number is GIXT00000000.

## Author Contributions

HW conceived the research plans. HW and DL supervised the experiments. YZ performed most of the experiments. HG and ZC provided technical assistance to YZ. HW and YZ designed the experiments and analyzed the data. HW conceived the project. YZ wrote the manuscript with contributions of all the authors. HW and DL supervised and complemented the writing. All authors contributed to the article and approved the submitted version.

## Conflict of Interest

The authors declare that the research was conducted in the absence of any commercial or financial relationships that could be construed as a potential conflict of interest.

## Supplementary Material

Supplementary Figure 1 PCR results of the *TaNAC069* gene. M, Marker 2000; 1, *TaNAC069*.

Supplementary Figure 2 Cloning of the *TaNAC069* promoter. M, Marker 2000; 1, *TaNAC069* promoter.

Supplementary Figure 3 *Cis*-acting element prediction of the *TaNAC069* putative promoter. The nucleotides were numbered relative to the transcription start site (+1) by the black bold font. The partial sequence of the *TaNAC069* gene is indicated by the black bold font. Other important putative *cis*-acting elements were identified using PlantCARE and the PLACE database. These *cis*-acting elements are underlined and presented with different-colored background.

Supplementary Figure 4 PCR products of the pBI121-pTaNAC069 plasmid. M, Marker 2000; 1–3, PCR products of the pBI121-pTaNAC069 plasmid.

Supplementary Figure 5 Screening of a yeast one-hybrid library and obtaining upstream transcription factors. **(A)** Restriction digestion of the bait vector pAbAi-*pTaNAC069*. M, Marker 5000; 1, pAbAi-*pTaNAC069* plasmid; 2, digestion with *Hind*III and *Kpn*I. **(B)** Identification of bait–yeast strains by yeast colony PCR. M1, Marker 2000; M2, Marker 5000; 1–2, positive control *p53*; 3–4, PCR products of the bait vector pAbAi-*pTaNAC069* transformed into the yeast genome. **(C)** The results of positive yeast clones in SD/-Leu/AbA (0–200 ng/ml) solid medium. **(D)** The partial PCR identifications of positive yeast clones by yeast one-hybrid assays. M_3_, Marker 2000.

Supplementary Figure 6 Screening of a yeast two-hybrid library and obtaining interacting targets of TaNAC069. **(A)** Self-activation detection of the bait. **(B)** Toxicity of the bait. **(C)** The partial PCR identifications of positive yeast clones by yeast two-hybrid assays. M, Marker 2000.

Supplementary Figure 7 Statistics of uredinias. Statistics of uredinia on 2-cm inoculated leaf segments at 14 dpi; six segments were taken from each sample. Data are means ± standard errors of three independent experiments. Different samples were assessed using SSPS. ^∗^*p* < 0.05; *n* = 3.

Supplementary Table 1 Primers used in this study.
