# Supplementary figures and images for: Functional Analysis of Wheat NAC Transcription Factor, TaNAC069, in Regulating Resistance of Wheat to Leaf Rust Fungus
Source: Front Plant Sci. 2021 Mar 15;12:604797. doi: 10.3389/fpls.2021.604797 (PMC8005738; doi:10.3389/fpls.2021.604797)

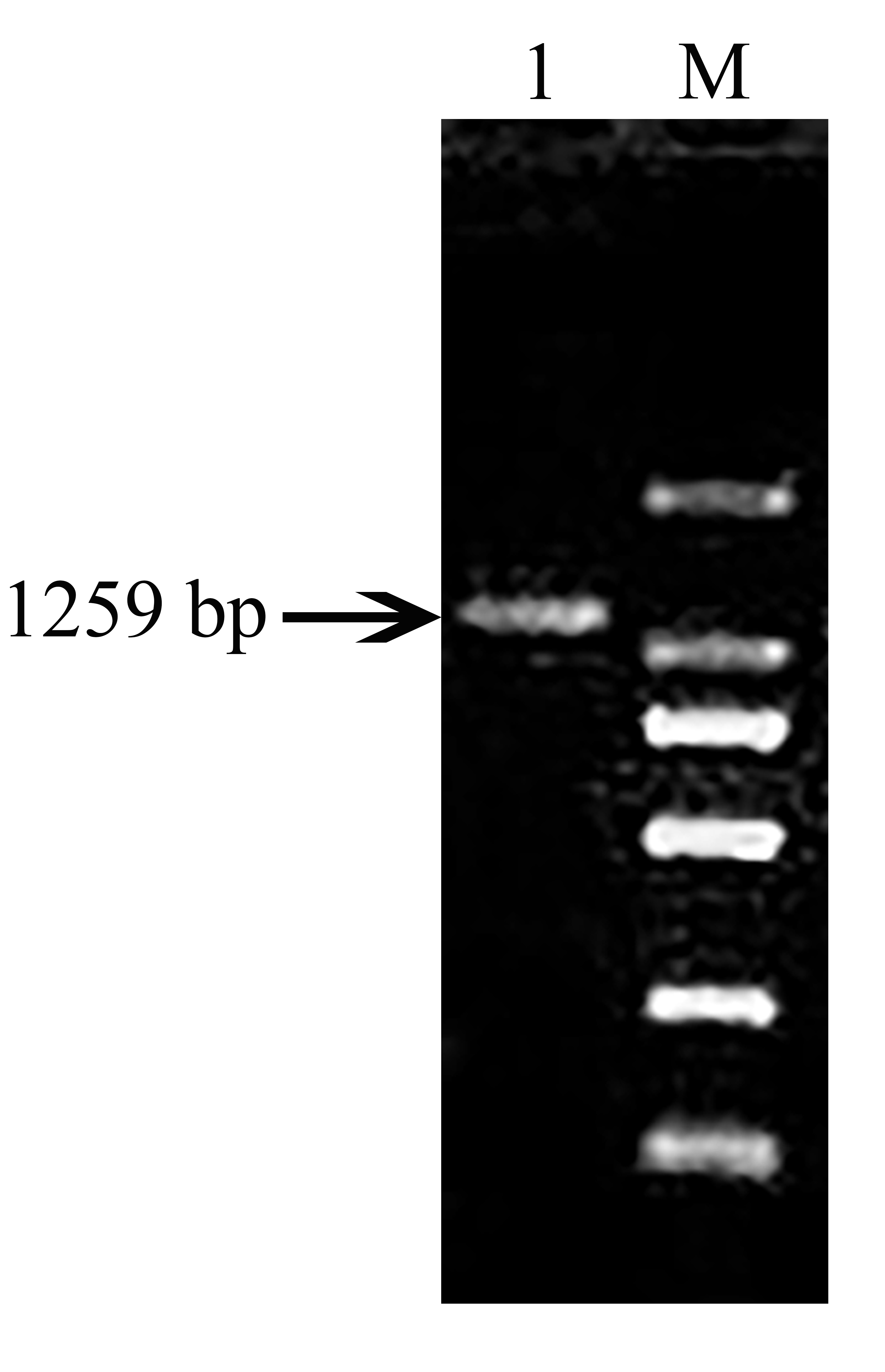

Supplement: Supplementary Figure 1 — PCR results of the TaNAC069 gene. M, Marker 2000; 1, TaNAC069. [file Image_1.TIF]

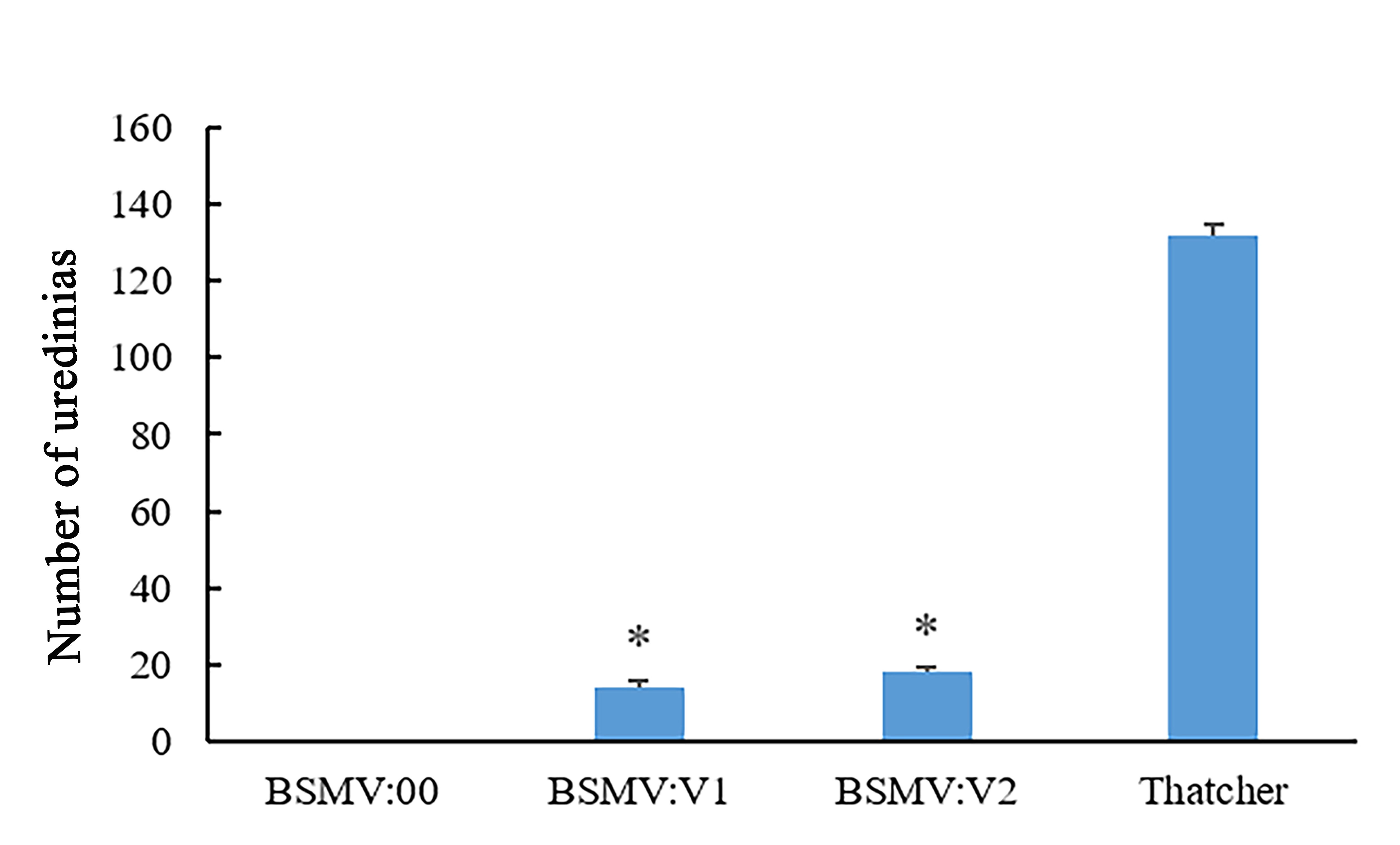

Supplement: Supplementary Figure 2 — Cloning of the TaNAC069 promoter. M, Marker 2000; 1, TaNAC069 promoter. [file Image_2.TIF]

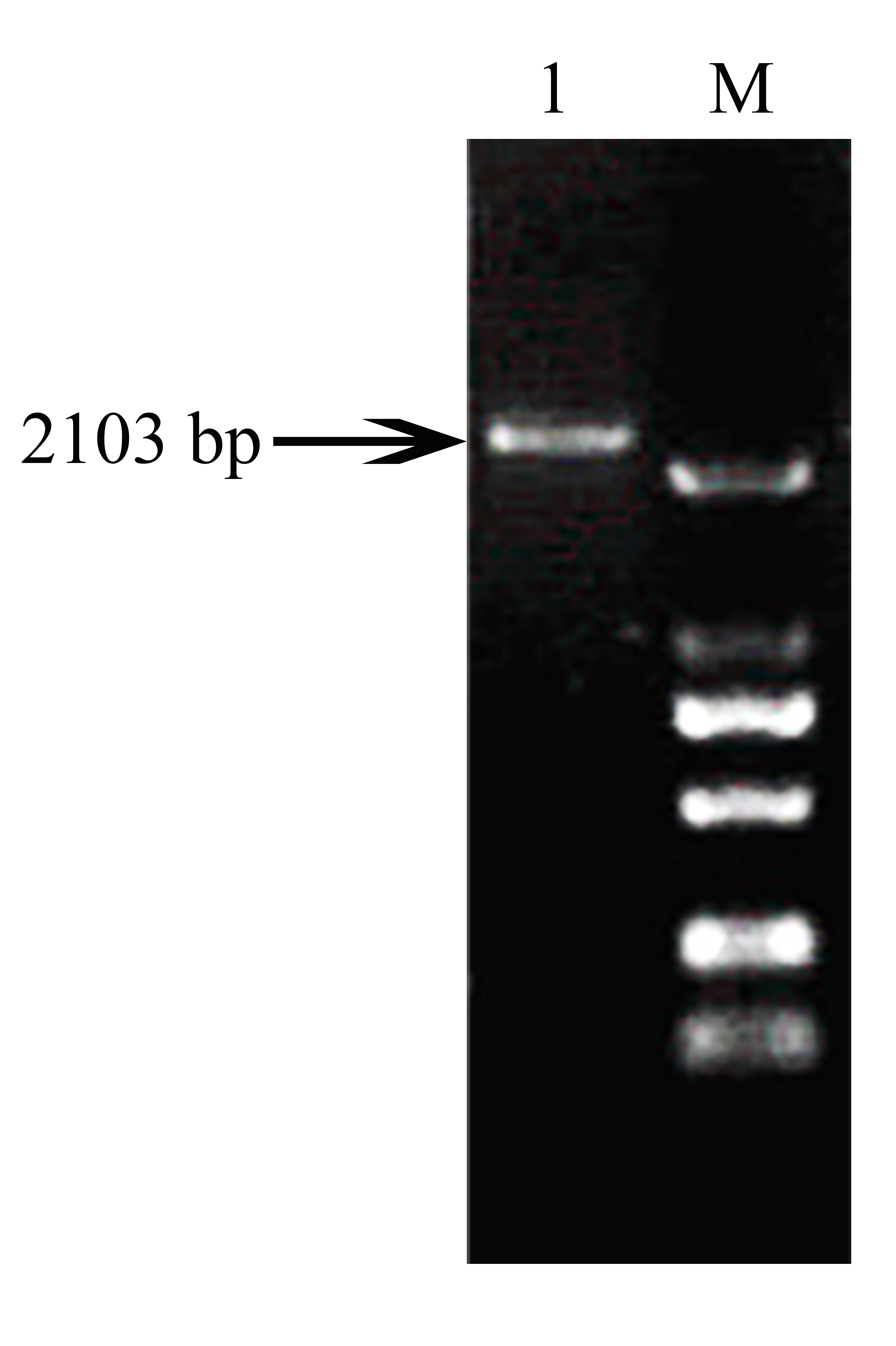

Supplement: Supplementary Figure 3 — Cis-acting element prediction of the TaNAC069 putative promoter. The nucleotides were numbered relative to the transcription start site (+1) by the black bold font. The partial sequence of the TaNAC069 gene is indicated by the black bold font. Other important putative cis-acting elements were identified using PlantCARE and the PLACE database. These cis-acting elements are underlined and presented with different-colored background. [file Image_3.TIF]

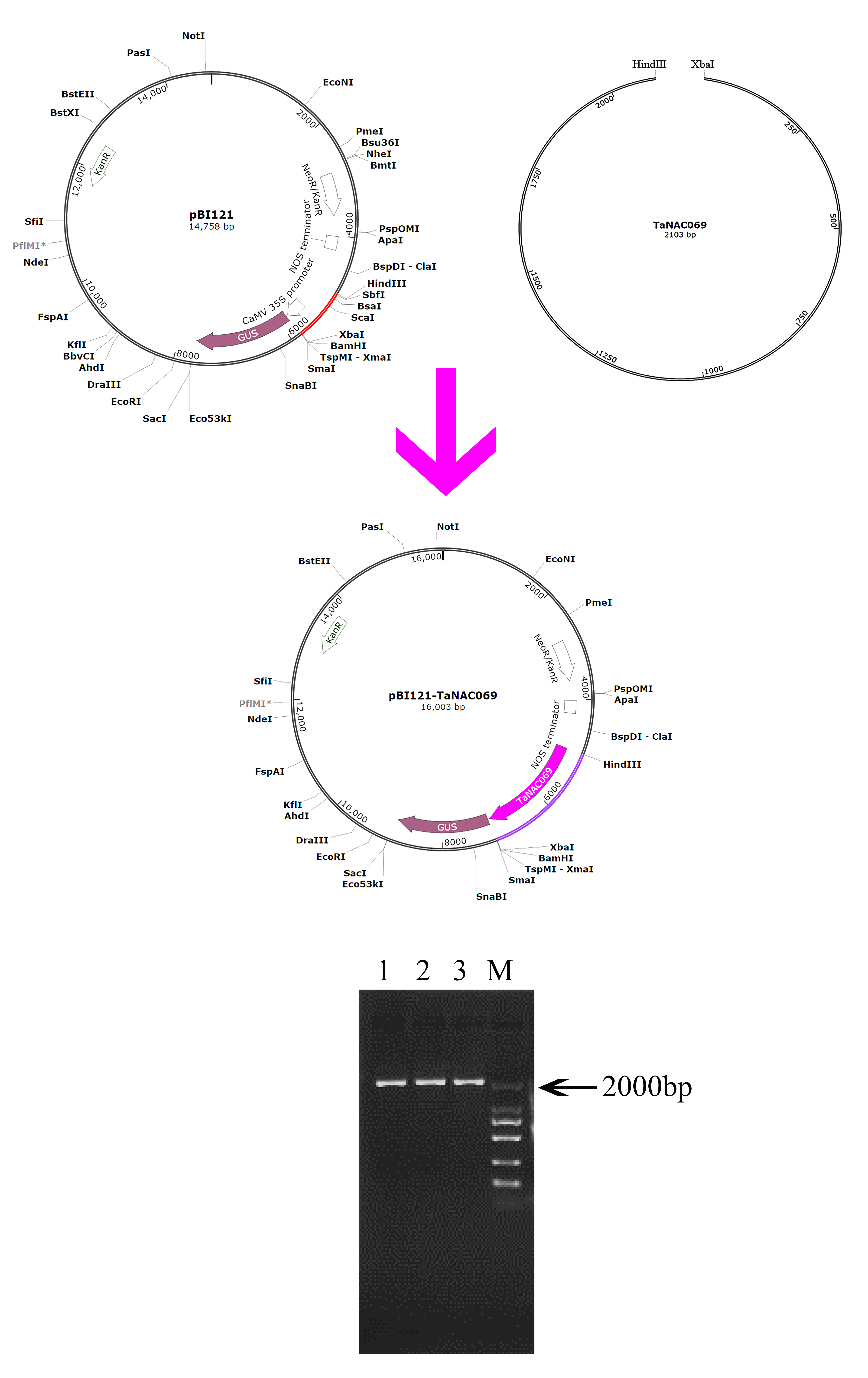

Supplement: Supplementary Figure 5 — Screening of a yeast one-hybrid library and obtaining upstream transcription factors. (A) Restriction digestion of the bait vector pAbAi-pTaNAC069. M, Marker 5000; 1, pAbAi-pTaNAC069 plasmid; 2, digestion with HindIII and KpnI. (B) Identification of bait–yeast strains by yeast colony PCR. M1, Marker 2000; M2, Marker 5000; 1–2, positive control p53; 3–4, PCR products of the bait vector pAbAi-pTaNAC069 transformed into the yeast genome. (C) The results of positive yeast clones in SD/-Leu/AbA (0–200 ng/ml) solid medium. (D) The partial PCR identifications of positive yeast clones by yeast one-hybrid assays. M3, Marker 2000. [file Image_5.TIF]

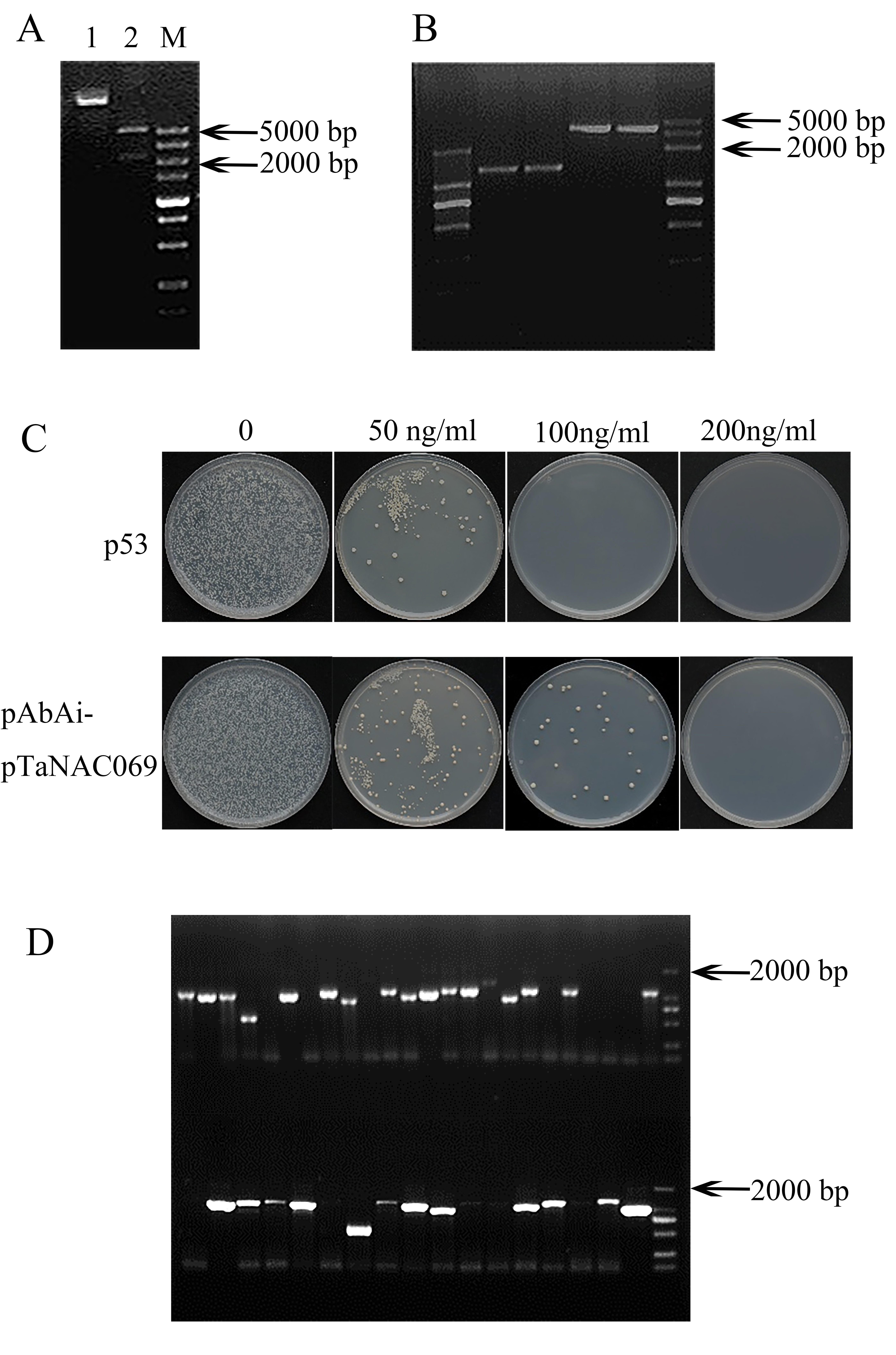

Supplement: Supplementary Figure 6 — Screening of a yeast two-hybrid library and obtaining interacting targets of TaNAC069. (A) Self-activation detection of the bait. (B) Toxicity of the bait. (C) The partial PCR identifications of positive yeast clones by yeast two-hybrid assays. M, Marker 2000. [file Image_6.TIF]

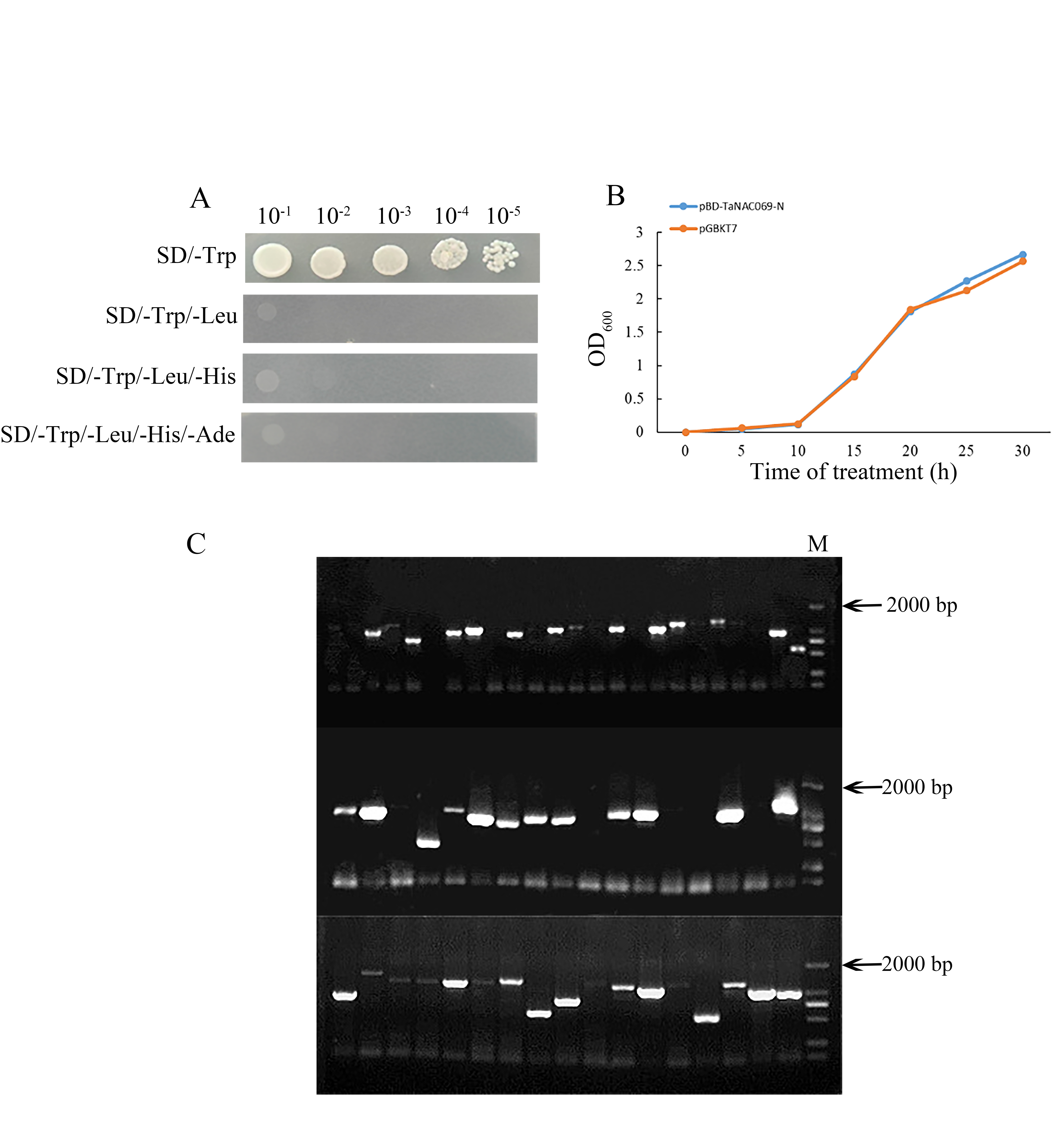

Supplement: Supplementary Figure 7 — Statistics of uredinias. Statistics of uredinia on 2-cm inoculated leaf segments at 14 dpi; six segments were taken from each sample. Data are means ± standard errors of three independent experiments. Different samples were assessed using SSPS. ∗p < 0.05; n = 3. [file Image_7.TIF]
