# Supplementary material for: Functional Analysis of Wheat NAC Transcription Factor, TaNAC069, in Regulating Resistance of Wheat to Leaf Rust Fungus
Source: Front Plant Sci. 2021 Mar 15;12:604797. doi: 10.3389/fpls.2021.604797 (PMC8005738; doi:10.3389/fpls.2021.604797)
Supplement: Supplementary Table 1 — Primers used in this study. [file Table_1.DOCX]

**Supplementary table 1 Primers used in this study**

| Primer | 5'- 3' sequence |
| --- | --- |
| TaNAC069-F | 5'- CAGCAGCCCACACCAAC -3' |
| TaNAC069-R | 5'- GTTCTTATCGGTCGGTCGTTC -3' |
| qTaNAC069-F | 5'- AGAACAACCACACCGTATCG -3' |
| qTaNAC069-R | 5'- TAGTGTAGCGTTTGTTGCCA -3' |
| pBD-TaNAC069-F1 | 5'- CGCCATATGGCGATGCCAATGGGCAGCAGCGCCGC -3' |
| pBD-TaNAC069-R1 | 5'- CCGGAATTCCGGGAGGCGGTACTCGTGCATGAT -3' |
| pBD-TaNAC069-F2 | 5'- CGCCATATGGCGACCGCAGCCGACGACCGGACC -3' |
| pBD-TaNAC069-R2 | 5'- CCGGAATTCCGGCATGTGCAGCTGCTGGCTGAG -3' |
| pL-TaNAC069-F1 | 5'- GGTACCATGCCAATGGGCAGCAGCGCCGC -3' |
| pL-TaNAC069-R1 | 5'- ACTAGTCATGTGCAGCTGCTGGCTGAG -3' |
| V- TaNAC069-F1 | 5'- ATATTAATTAATCGCAGAACAACCACACC -3' |
| V-TaNAC069- R1 | 5'- TATGCGGCCGCTAGTGTAGCGTTTGTTGCCA -3' |
| V-TaNAC069- F2 | 5'- ATATTAATTAAGCAACAAACGCTACACTAT -3' |
| V-TaNAC069 -R2 | 5'- TATGCGGCCGCTGGCTGAGGAACGGATGGA -3' |
| TaSOD-F | 5'- GGCTCTCCAAGGTCGTGT -3' |
| TaSOD-R | 5'- GGGTTGCCGTTGTTGTAG -3' |
| TaCAT-F | 5'- GCCTGTGTTTTTTATCCGAGA -3' |
| TaCAT-R | 5'- CAGGTGCCTCCAACAGTAACA -3' |
| TaNOX-F | 5'- AGATGGAGGAAGAGGAGGATA -3' |
| TaNOX-R | 5'- CGTAAACGCTTGTGAGGTAGT -3' |
| qTcLr19PR1-F | 5'- CAATAACCTCGGCGTCTTCATC -3' |
| qTcLr19PR1-R | 5'- ATTTACTCGCTCGGTCCCTC -3' |
| qTcLr19PR2-F | 5'- CAACGAGAACCAGAAGGACAGC -3' |
| qTcLr19PR2-R | 5'- TACGGACGGACATACGGACACT -3' |
| qTcLr19PR5-F | 5'- GAACAACTGCGGCTCCAC -3' |
| qTcLr19PR5-R | 5'- CTACCGCCATTGAAGGAGC -3' |
| pTaNAC069-F | 5'- GCTCAAGAACACCTCAACCT -3' |
| pTaNAC069-R | 5'- ACTTGTAGATGTTGACCTCG -3' |
| pBI121-pNAC069-F | 5'- CTATGACCATGATTACGCAGCTCAAGAACACCTCAACCTCAGGAG -3' |
| pBI121 -pNAC069-R | 5'- ACTGACCACCCGGGGATCCTGGCTGGCGATGGCGAGCA -3' |
| PCamA -pNAC069-F | 5'- AAGCTTGCTCAAGAACACCTCAACCT -3' |
| pCamA- pNAC069-R | 5'- GGTACCGGCTGGCGATGGCGAGCAGGT -3' |
| pAbAi-pNAC069-F | 5'-AAGCTT GCTCAAGAACACCTCAACCT -3' |
| pAbAi -pNAC069-R | 5'- CTGGTACCGGCTGGCGATGGCGAGCAGGT -3' |
| qTaCP5-F | 5'- CCTCAGCGTACAGCATTTCGA -3' |
| qTaCP5-R | 5'- GGGTTGGAGTCGGTGAAGGAG -3' |
